# Need of orthogonal approaches in neurological disease modeling in mouse

**DOI:** 10.3389/fnmol.2024.1399953

**Published:** 2024-05-02

**Authors:** Linda Bossini, Alessandro Sessa

**Affiliations:** ^1^Neuroepigenetics Unit, Division of Neuroscience, IRCCS San Raffaele Scientific Institute, Milan, Italy; ^2^“Vita e Salute” San Raffaele University, Milan, Italy

**Keywords:** mouse model, genetic engineering, neurodevelopmental disorders, neurodegenerative disorders, reversibility

## Abstract

Over the years, advancements in modeling neurological diseases have revealed innovative strategies aimed at gaining deeper insights and developing more effective treatments for these complex conditions. However, these progresses have recently been overshadowed by an increasing number of failures in clinical trials, raising doubts about the reliability and translatability of this type of disease modeling. This mini-review does not aim to provide a comprehensive overview of the current state-of-the-art in disease mouse modeling. Instead, it offers a brief excursus over some recent approaches in modeling neurological diseases to pinpoint a few intriguing strategies applied in the field that may serve as sources of inspiration for improving currently available animal models. In particular, we aim to guide the reader toward the potential success of adopting a more orthogonal approach in the study of human diseases.

## Introduction

All cells within a complex multicellular organism retain the same genetic information. The regulation of gene expression is the crucial mechanism to interpret and utilize such information and create the overarching diversity of cell types composing those organisms. The interplay of various intrinsic and extrinsic factors influences gene expression. They can induce either expression or silencing at specific time points, ultimately dictating cellular identity, morphology, and function (Savulescu et al., 2020) both during embryonic development and throughout subsequent adult life (Pope and Medzhitov, 2018).

In the central nervous system (CNS), neuronal development unfolds over a series of intricate processes encompassing proliferation, migration, differentiation, synaptogenesis, and pruning, to ensure the formation of functional neuronal connectivity. These biological events are subjected to a tight regulation by specific genetic programs, which operate following precise spatiotemporally-defined fashions (Subramanian et al., 2020). Even subtle alterations to these programs can disrupt the proper assembly and maturation of neuronal circuits, thereby triggering neurological pathology (Griffin et al., 2022).

As a result, decoding the functions of a gene of interest, or its pathological variants, in relevant biological landscapes represents a major challenge. To this endeavor appropriate experimental genetic tools, capable of providing a commensurate level of complexity in terms of controlled gene expression, are fundamental. Employing various scales of investigation, such as cell-type or age-specific approaches, can yield valuable insights into previously unknown gene functions and their roles in disease development or progression. Understanding the intricacies of these genetic programs and how they are disrupted in disease states is essential for uncovering the underlying mechanisms of neurological disorders and devising effective therapeutic interventions.

## Lost in “translation”: challenges of animal disease modeling

A spectrum of technologies, spanning from conventional to more advanced *in vitro* and *in vivo* systems, alongside computational modeling, has been explored in the pursuit of elucidating the molecular underpinnings of finely orchestrated processes, all with the overarching goal of reliably recapitulating human diseases (Paşca et al., 2014; Birey et al., 2017; Jönsson et al., 2020; Pomeshchik et al., 2020; Susaimanickam et al., 2022; Li et al., 2023; Meng et al., 2023). In this direction, across many years, mouse models emerged as the cardinal tool for investigating disease mechanisms, progression and potential therapeutic strategies (Lunev et al., 2022).

Nonetheless, despite the advantages and versatility of animal models, their translational utility has faced increasing scrutiny in recent years. One of the main concerns arises from the failure of several clinical trials, despite the promising results obtained from preclinical studies conducted in, mainly, murine models (Bespalov et al., 2016; Seyhan, 2019; Marshall et al., 2023).

Several factors contribute to the translational gap between preclinical animal studies and clinical trials including species differences, model fidelity and disease heterogeneity. For instance, while animal models may recapitulate certain aspects of human diseases, they often fail to fully replicate the complexity of human physiology, and therefore pathology. In addition, heterogeneity should be taken into considerations. In the first place, many human diseases are themselves characterized by significant heterogeneity in terms of etiology, pathophysiology and clinical presentation as exemplified by incomplete penetrance and variable expressivity (Kingdom and Wright, 2022). So it is clear that such heterogeneity can hardly be modeled by a unique disease model. Moreover, genetic (e.g., mouse genetic backgrounds) and environmental factors (e.g., experimental conditions) also may impact on the fidelity of the model (Robinson et al., 2019; Georgiou et al., 2022).

Despite these inherent limitations, animal models have proven to be invaluable tools over the years. Thus, it's crucial to acknowledge and eventually minimize these constraints while exploring approaches to instead maximize the potential of the models. Creating a unique, informative, and robust model for a disease is often hardly feasible. Instead, applying an orthogonal approach, e.g., based on the integration of independent methodologies or model systems to address the same biological topic, may result a more illuminating strategy. The current trend in the animal modeling field is indeed evolving toward a “network” approach, in which multiple types of models are employed, and further experiments are redesigned based on evidence gained from the other models (Pasko et al., 2023). This integrated manner provides complementary insights and validation of findings, enhancing the understanding of complex processes and disease mechanisms, thus facilitating the translation of research findings into clinical applications.

Often the investigation of the pathological consequences of disease-causing gene variants begins with modeling the extreme conditions: either total deletion or overexpression of the gene of interest. Alternatively, since in many complex genetic diseases, there exists a well-established inverse relationship between disease-causing genetic variants and the severity of the corresponding phenotype, researchers often exploit these rarer variants with large effect sizes as a practical starting point for delving into the investigation of the disease's neurobiology to delineate the causal pathways (Amanat et al., 2020; Gordon and Binder, 2023). On this line, constitutive genetically engineered mouse models represent the standard condition to approach the study of the effects of genetic mutations. In fact, they generally provide a broad overview of the disease and its most severe manifestations (Dow, 2015). However, they may fail to represent ideal platform for dissecting relevant phenotypes in depth, especially in the context of neurodevelopmental and neurodegenerative disorders.

These models frequently exhibit such severe manifestations that may obscure the underlying subtle events contributing to pathology. One exemplificative case is the embryonic lethality or reduced survival of genetically modified animals, which make challenging to study the effects of these mutations in mature organs up to adult organisms. Additionally, the intricate and dynamic processes at the basis of neurological disorders may only become evident at specific levels of analysis (e.g., certain developmental stages, cell populations, higher-order brain processes, network connectivity, etc.) (Gordon and Binder, 2023) or may only manifest when different pathological insults or risk factors converge, as suggested by dual-hit hypotheses (Zhu et al., 2007; Rietdijk et al., 2017; Guerrin et al., 2021).

Similarly in the neurodegenerative disorders' scenario, the experimental design of the most commonly used models was indeed to recapitulate the hallmarks of the disease (e.g., cell death, protein aggregation, inflammation etc.), often exacerbating them (e.g., acute induction at extraordinary high levels), to make immediately accessible the study of the desired phenotype. However, these models do not respect the gradual development of the pathology, corresponding symptomatology and, likely, associated underlined biological processes. For this same reason, conventional models, since do not allow for *ad hoc* modulation of gene expression at specific stages, do not represent the ideal platform for studying adult-onset neurodegenerative disorders as well, where precise temporal control over the expression of disease-associated genes should be pursued to align with the natural time course of the disease.

*To contextualize our premises, let's frame our discussion with a hypothetical neurological condition X arising from the accumulation, and subsequent aggregation, of a mutated protein X. While the exact mechanisms are yet unknown, this buildup is believed to disrupt the function of either excitatory or inhibitory neurons. This results in an altered balance between excitation and inhibition, ultimately leading to cognitive and motor deficits*.

**Figure 1:**
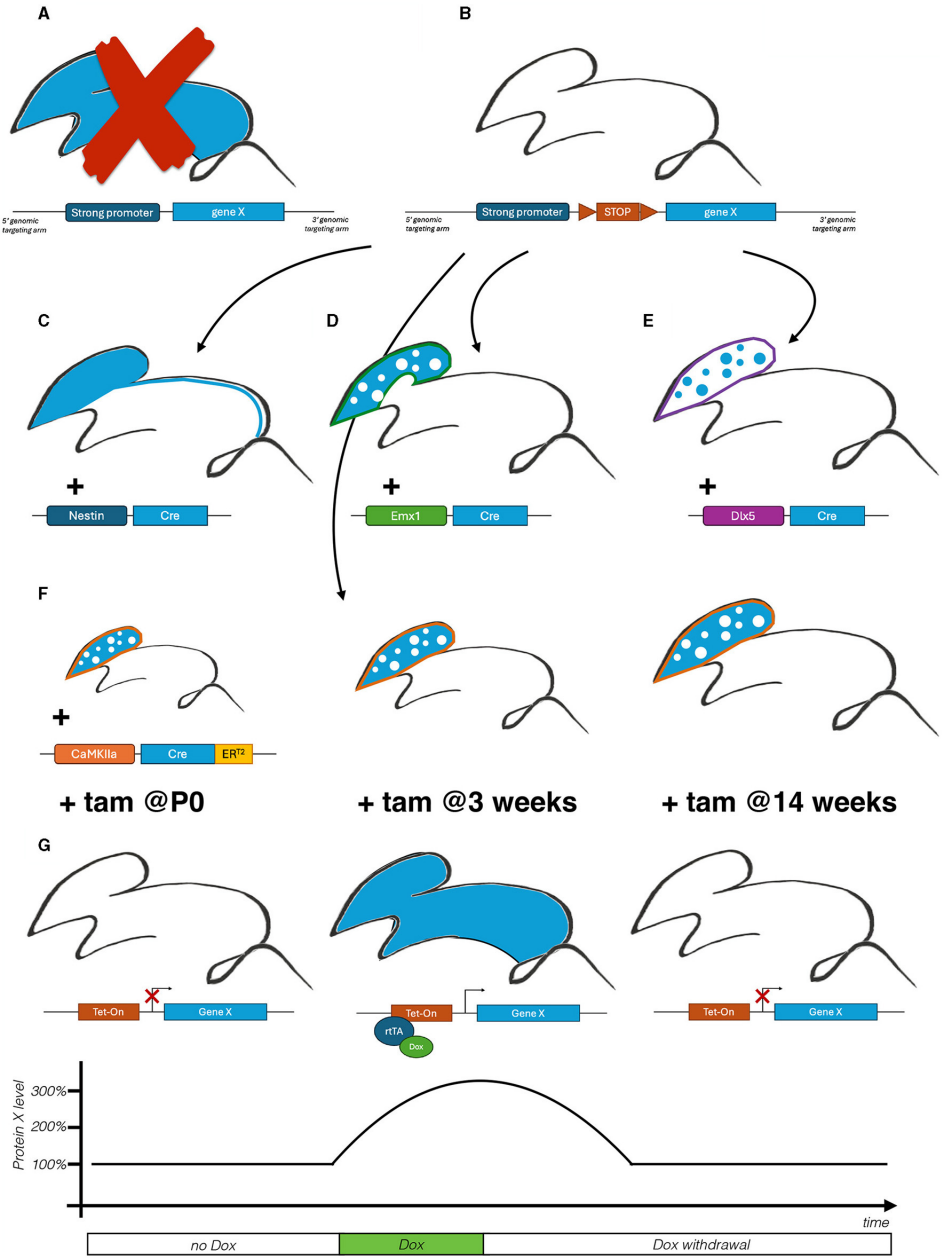
Schematic representation of an illustrative orthogonal approach applied on the investigation of a fictitious X disease. **(A)** Generation of a transgenic mouse model to induce constitutive gene X overexpression. **(B)** Generation of a conditional Cre-dependent knock-in mouse line harboring an additional copy of the gene X under the control of a floxed STOP cassette for on demand induction of pathological protein accumulation. **(C)** Generation of a conditional model overexpressing gene X in the entire CNS, including the spinal cord. **(D, E)** Generation of a conditional model overexpressing gene X in excitatory **(D)** and inhibitory neurons **(E)** to explore their contribution to the pathological phenotype. **(F)** Generation a conditional model overexpressing gene X in excitatory neurons upon Tamoxifen (tam) induction, at three postnatal timepoints, to explore their temporal susceptibility to the pathological insult. **(G)** Generation of a tet-inducible reverse transactivator (rtTA)-dependent conditional model. Initial administration of Doxycycline (Dox) induces protein X accumulation, while following Dox withdrawal restores protein levels to mimic various dynamics of therapeutic interventions.

*For an initial investigation, protein accumulation is experimentally mimicked by the generation of constitutive transgenic mouse model overexpressing the gene X (Figure 1A). Unfortunately, the new line is characterized by a high mortality rate, making the study of the pathological underpinnings hardly feasible. This outcome opens up the necessity of exploring an alternative strategy to model the condition*.

To holistically understand the etiopathogenesis of a disease, both the cellular origin and the precise timing of its pathogenesis are to be carefully considered. In the framework of neurological disorders, this means identifying the critical time window when the CNS becomes vulnerable the most to the pathogenic insult, either protein loss or accumulation, and where this happens. Instead, in a therapeutical perspective, to determine the “point of no return,” so when the organ is no longer able to recover from that challenge.

## Exploring space and time: novel insights from animal models

While constitutive models are useful for generating experimental conditions with readily observable phenotypes, thus serving as starting point of investigation, conditional genetic engineering may provide higher level of information.

Conditional knock-out or knock-in models, used for an alternative but targeted explorative approach, have revolutionized our ability to ask specific biological questions with greater precision and control. In this framework, the Cre/loxP recombination system represents the cornerstone of conditional genetic manipulation in mice, including gene deletion, insertion, inversion, or translocation. Applied for the first time *in vivo* by Rajewsky and Marth in 1994, this “simple” yet powerful tool soon became indispensable for interrogating the genetic and molecular basis of diseases (Gu et al., 1994; Rajewsky et al., 1996; Heldt and Ressler, 2011).

The system facilitates the controlled insertion of desired genetic modifications by exploiting two key components: the strategical incorporation of loxP sequence recognition sites to flank (“floxed”) a DNA sequence (e.g., gene of interest, STOP cassette) and the regulated expression of Cre recombinase under the control of a specific promoter. Therefore, the adaptable nature of the Cre/loxP system provides the sought flexibility in experimental design, making it invaluable for uncovering several information.

*To overcome the limitation of the previous constitutive model, a novel conditional allele been generated to modulate ad hoc gene X expression and the consequential protein accumulation. The novel line carries an additional gene X copy preceded by a floxed STOP cassette (Figure 1B). Given the CNS-related phenotype, the disease has been mimicked crossing the new line with the Nestin-Cre, active in neural progenitors (Figure 1C). The resulting mutant recapitulates disease-associated neurological phenotypes, including cognitive (e.g., anxiety, memory) and motor deficits (e.g., balance, coordination)*.

This approach was nicely exemplified by Li J. et al. (2022) who employed various Cre lines to investigate *AUTS2*'s functions, a gene implicated in autism spectrum disorders (ASDs), within the brain. Since a constitutive knock-out model resulted in embryonic lethality, the authors generated *Auts2* floxed mice subsequently crossed with different Cre lines to conditionally disrupt *Aust2* at distinct developmental stages and in specific forebrain regions. For instance, using Cre lines with region-specific expression patterns enabled them to discern *Auts2*'s role in different phases of dentate gyrus (DG) specification. This temporally-dictated analysis was fundamental to confirm *Auts2* function for DG development only at early postnatal stage, highlighting its critical involvement as transcription repressor in a previously unknown mechanism for neural cell migration. To specifically analyze the gene's function during the postnatal phase, they also took advantage of viral tools for delivering the Cre recombinase. This approach not only offered additional spatial control, through local injections, but also provided temporal control, via the timing of injection. This strategy proved advantageous in avoiding potential compensatory mechanisms triggered by gene knockdown throughout development.

*To further deepen the pathological basis of disease X, the Emx1-Cre and the Dlx5/6-Cre have been employed to induce accumulation in either excitatory or inhibitory neurons respectively (Figures 1D, E). Notably, only the excitatory-restricted mutants present few of the neurological phenotypes*.

The injection of virally-delivered Cre, using replication-deficient adeno-associated (AAV) or lentiviral (LV) vectors, serves as a potent tool for controlling the expression of the gene of interest (Heldt and Ressler, 2011; Lunev et al., 2022). This approach increases the flexibility of the Cre/loxP system to adapt to various experimental needs, including the exploration of gross structural changes, as well as consequential interrogation of functional aspects, such as neuronal circuit activity, in greater detail (Hui et al., 2022). In a recent study by Yonan and Steward (2023), to examine the deletion of *Pten* in fully mature neurons, a gene implicated in ASDs in humans, an AAV-delivered Cre has been unilaterally injected in the *Pten* floxed model. Specifically, the viral particles have been introduced directly into the DG to induce a focal deletion, while maintaining *Pten* expression in the cells of origin of the input circuits. This strategy is noteworthy because, unlike constitutive models, it allows for investigation of the direct primary effects of the gene, without interferences resulting from gene suppression in other connected regions. This approach revealed how *Pten* suppression at the level of the DG triggers synapse formation, independently of PTEN activity in the presynaptic cells.

Viral delivery is an excellent option for achieving focal expression, or deletion, of a gene of interest, precisely in space and time. In adult animals, viruses can be delivered through various methods, such as tail vein injections for systemic delivery, intrathecal or stereotaxic injections for a more localized delivery. On the other hand, for perinatal expression, intracerebroventricular or retro-orbital injections may be employed. Nevertheless, each of these surgical procedures requires a different level of technical expertise.

Over the years, various advancements of the original recombination system have emerged to achieve even finer spatiotemporal comprehension (Shcholok and Eftekharpour, 2023). These include the tamoxifen-inducible Cre-ER^T2^ system (Belteki et al., 2005; Heldt and Ressler, 2011; Hui et al., 2022; Kellogg et al., 2023), photoactivatable Cre recombinases (Yoshimi et al., 2021; Li et al., 2022), Cre/Dre dual recombinase systems (Kouvaros et al., 2023) and split Cre systems (Khoo et al., 2020; Kim et al., 2021), among others. Some of these configurations are of extreme help to easily explore the time and the space, inducing the pathological insult within cell types at various embryonic and postnatal timepoints with high specificity.

One effective method involves the tamoxifen-inducible Cre-ER^T2^ system coupled with the plethora of promoters available today to regulate its induction (Hayashi and McMahon, 2002; Shcholok and Eftekharpour, 2023). In this system, the Cre recombinase is fused to a modified estrogen receptor ligand-binding domain (ER^T2^) that responds to tamoxifen, a synthetic estrogen analog. Once expressed in the cell, this Cre-recombinase variant remains in the cytoplasm without exerting any activity till the administration of the drug, that triggers the translocation of the Cre into the nucleus to perform its activity.

Sonzogni et al. (2019) leveraged the flexibility offered by this inducible system in the context of Angelman syndrome (AS), a severe neurodevelopmental disorder, to investigate the role of UBE3A, a ubiquitin ligase amply implicated in brain development and maturation. Since previous studies predominantly focused on UBE3A critical functions in early brain development, the researchers aimed to understand its role after normal brain development. To address this issue, they generated a conditional mouse model which was crossed with either a constitutive Cre line to induce *Ube3a* embryonic deletion, establishing a baseline for behavioral phenotypes, or the CreER^T2^ to trigger protein loss on demand, e.g., after early brain development and later in adulthood. This system proved crucial in confirming UBE3A's role in normal brain development, specifically its role in shaping neuronal connectivity (e.g., synapse development) and its subsequent involvement in motor coordination and cognitive functions (Greer et al., 2010; Khatri and Man, 2019). This further delineates embryonic and early postnatal stages as the critical window of vulnerability of the brain to UBE3A protein loss. Notably, these findings also suggest that early, transient UBE3A reinstatement is likely to prevent most adverse phenotypes, underscoring the importance of initiating gene reactivation therapies early in life.

*To define the critical window of vulnerability of excitatory neurons in X disease, a CaMKIIa-ER^T2^-Cre is employed to induce protein X accumulation at differential timepoints (Figure 1F): at birth (P0), juvenile (3 weeks of age) and adult mice (14 weeks). This longitudinal approach allows to identify early postnatal stage as the most susceptible to damages ensuing protein accumulation*.

This chemically-inducible system can be utilized also to generate improved animal models, e.g., for studying disease stages that were previously inaccessible. A study in the context of Multiple System Atrophy (MSA), an adult-onset synucleinopathy characterized by the presence of cytoplasmic inclusions in oligodendrocytes, represents a nice example. Given the adult-onset nature of the disease, to induce α-synuclein (Syn) expression only in adulthood, Tanji et al. generated a novel floxed Syn knock-in mouse line, which was subsequently crossed with a Cre-ER^T2^ driver line under the control of an oligodendrocyte-restricted promoter (Tanji et al., 2019). This enabled the generation for the first time of a model to investigate the effects of Syn expression on MSA pathophysiology specifically during the initial stages of disease progression, avoiding any latent disturbance or compensatory effect due to sustained promoter activity during the developmental stages. Given the significance of early treatment in slowing MSA progression, this model offers the unique opportunity to enhance understanding of the events driving initial disease progression and to develop novel therapeutics for MSA.

While inducible systems, like virally-delivered or chemically-inducible Cre-dependent ones, offer valuable insights into subtle pathogenic mechanisms, their acute induction represents an artificial deviation from the natural time course of the disease. However, if carefully designed, conditional knockout or knock-in models offer an exclusive platform that can be smartly utilized to systematically address many of the biological questions arising when investigating a pathological condition. This allows researchers to select the desired level of complexity, such as specific cellular populations, brain regions, or temporal stages, in a more controlled and physiologically relevant manner. Therefore, while conditional models may not fully recapitulate the complexity of human diseases, they offer unique advantages for investigating in depth fine pathogenic mechanisms with a level of precision that is often not unattainable with constitutive models.

## Unveiling reversibility: flipping the paradigm

By providing precise spatial and temporal control over the disruption or overexpression of a gene, Cre-based systems are invaluable tools for addressing specific biological questions. Nonetheless, a primary constraint lies in the irreversibility of the recombination once Cre activation, whether constitutive or inducible, triggers it. The capability to first induce and subsequently halt the pathological challenge, thus emulating a potential treatment, yields insights into two critical facets: the reversibility of disease symptoms and the temporal necessities for therapeutic intervention.

For such reversible temporal regulation of gene expression, the Tetracycline (Tet)-inducible binary system could be considered. The Tet-On and Tet-Off systems allow to switch gene expression on or off respectively, in response to the presence or absence of tetracycline analogs (e.g., doxycycline). This system requires the combination of two components *in trans*: the tet-inducible transactivator (tTA) or reverse transactivator (rtTA), artificial transcription factors under the control of a tissue-specific promoter, and a modified tet-responsive element, which is activated upon binding of the tTA/rtTA (Dogbevia et al., 2015). Therefore, this system can easily achieve a spatiotemporal regulation similar to the one of the Cre/loxP system, implementing reversibility as an additional layer of control (Belteki et al., 2005).

Although tTA-dependent alleles corresponding to various proteinopathies already exist, one of the significant limitations of current mouse models for neurodegenerative diseases, is their failure to properly mimic the disorder's time course (Koller et al., 2022). For example, in Alzheimer's disease models, clinical signs often manifest earlier than in the corresponding human pathology, implying a need to delay the formation of the pathological amyloid plaques. Additionally, the limited availability of CNS-targeting tTA driver lines poses a challenge for regional or cell-type-specific studies. To address these limitations, Koller and colleagues proposed using a Cre-to-tTA converter allele to yield Cre-dependent expression of an existing Tet-regulated transgene (Koller et al., 2022). This system allows to draw from the extensive repertoire of Cre driver lines for precise spatial control, including spatiotemporal control when considering Cre-ER^T2^ drivers. This novel model was able to delay amyloid formation adapting it to the natural disease progression, while also restricting this process in specific cell types. This innovative combinatorial approach could be applied to other diseases, neurodegenerative or otherwise, for which tTA-dependent models already exist, maximizing the utility and versatility of these powerful systems.

Combination of both systems, the Cre/loxP with the Tet-O, can be exploited to further enhance control over transgene induction. An example was recently done by Li J. et al. The authors aimed to delineate the temporal requirements for TorsinA in normal motor function in a mouse model of DYT1 dystonia, a neurodevelopmental disease induced by a loss-of-function mutation in the corresponding *TOR1A* gene (Li et al., 2021). Previous studies focused on conditionally deleting TorsinA from various regions of the CNS, thus revealing the existence of a critical period of vulnerability of the brain to TorsinA loss (Tanabe et al., 2012). To describe this existing temporal interval, a mouse line enabling spatiotemporal control over the endogenous *Tor1a* gene was developed by inserting at the 5′ of the *Tor1a* start site a cassette containing a floxed STOP element followed by a tetracycline operator (Tet-O) in a Tet-On-like configuration to confer both Cre and tetracycline responsivity. The Cre system was used to restrict the defect in various brain regions to assess their temporal sensitivity to lack of TorsinA function, while the Tet system was employed to mimic a therapeutic restoration. Using this setting, the authors restored protein levels using three different temporal dynamics to simulate possible schedules of therapeutic intervention. This innovative approach allowed the delineation of a two-stage model for the disease: primary causative events directly related to TorsinA loss and therefore reverted by its restoration (stage 1) and the irreversible downstream molecular or circuit changes independent of TorsinA function (stage 2). Although not strictly physiological, this disease model has provided insights into pathogenic dynamics, with significant implications for the design and timing of effective therapeutic strategies.

The concept of implementing genetic reversibility in disease-mouse models is crucial for determining the possibility and extent of symptom reversion. This concept was pioneered by Guy et al. (2007), in the context of Rett syndrome, an ASD caused by mosaic expression of mutant copies of the X-linked *MECP2* gene in neurons. To understand whether the postnatal restoration of MeCP2 levels could reestablish the neuronal functionality, the scientists inserted a removable floxed STOP cassette in the endogenous mouse gene. The CreER^T2^ transgene was also present to control the reactivation of the gene using tamoxifen injection. This model demonstrated, for the first time, robust reversal of Rett pathology upon *Mecp2* restoration in both immature and mature adult animals, paving the way of the importance of implementing reversibility in the field of animal disease modeling.

As a postsynaptic scaffold protein, SHANK3 regulates synapse formation, function, and plasticity, hence its disruption in disease states results in synaptic defects and autistic-like behaviors. In the perspective of a restorative gene therapy, since increased levels of SHANK3 are connected to other neurological conditions (e.g., bipolar disorder) as well, Mei et al. (2016) proposed a novel conditional knock-in mouse model to investigate the temporal requirement of *Shank3* gene. In their work, they employed a Cre-dependent genetic switch (FLEx) strategy that allows *Shank3* expression upon its Cre-mediated inversion, to conditionally manipulate the endogenous gene (Schnütgen et al., 2003). By utilizing this reversible model, they highlighted an improvement in synaptic function upon impact of *Shank3* re-expression. However, only a few behavioral abnormalities were rescued, thus supporting the developmental origin of the autistic-like impairments due to *SHANK3* haploinsufficiency.

A reversible approach was similarly pursued by Mielnik et al. (2021) to explore the outcomes of counteracting N-methyl-D-aspartate (NMDA) receptor deficiency in the adult brain. Impaired NMDAR signaling has been coupled with various neurodevelopmental disorders over the years, leading to conditions such as intellectual disability, epilepsy, autism or schizophrenia. To assess the beneficial effects of a restorative treatment, they developed a mouse model where the loss-of-function allele of *Grin1*, the gene encoding for an NMDAR subunit, was induced via the insertion of a floxed STOP and thus restored to physiological levels upon Cre recombination. Rescued adult mice displayed robust improvements in cognitive functions, providing insights into the effectiveness of restoring NMDAR activity in adulthood.

Analogously in the context of Dravet syndrome, a severe epileptic encephalopathy primarily caused by haploinsufficiency of the *SCN1A* gene, Valassina et al. recently proposed a conditional knock-in mouse model enabling on-demand *Scn1a* reactivation, via a floxed STOP cassette removal. The authors successfully assessed the reversibility of the epileptic phenotype after disease onset (Valassina et al., 2022). Using this model, the same group recently investigated the effects of reactivating gene expression both perinatally and postnatally to define the critical window of vulnerability of the CNS to *Scn1a* loss, a crucial knowledge for future therapeutic interventions (Di Berardino et al., 2023).

*To demonstrate the reversibility of X disease, a Tet-On system has been employed to firstly induce and later suppress gene X overexpression to evaluate the effects of the reduction of protein levels (Figure 1G). Interestingly, while protein reduction at a late stage (14 weeks) was not able to revert the reported phenotypes, the recovery in juvenile mice (3 weeks) ameliorated both motor and cognitive impairments. Gathered together this data suggests for an early postnatal disease-modifying therapy*.

These approaches, as demonstrated in an increasingly number of neurological diseases, underscore the feasibility of therapeutically targeting their genetic root causes. However, not all diseases share this fortune, as demonstrated by Silva-Santos et al. in the context of Angelman syndrome (Silva-Santos et al., 2015). A conditional reversible mouse line, via the insertion of a floxed STOP cassette within *Ube3a* intron 3, has revealed the scarce efficacy of reactivating *Ube3a* in adult organisms, hence confirming the necessity of functional UBE3A at developmental stage.

## Discussion

Refining experimental models is essential for obtaining reliable results. Addressing significant biological questions, such as disease pathogenesis, is complex and, in our opinion must require confronting them from multiple perspectives. Tackle smaller aspects at a time, dividing this challenging endeavor, may return more complete answers for the questions the scientific community has. The use of conditional mouse lines harboring the gene of interest, and the different activating tools we have discussed, represent a major avenue to achieve this goal.

The precise spatiotemporal control afforded by these inducible systems enables investigation into the dynamic nature of biological mechanisms or pathological processes (e.g., primary events, compensatory mechanisms etc.) and disease progression over time. Leveraging these advanced genetic engineering tools facilitates the design of refined and physiologically relevant experimental conditions for studying neurodevelopmental and neurodegenerative disorders. This integrative “network” approach allows to uncover novel disease mechanisms and identify potential therapeutic targets that may not be apparent in more “classical” constitutive germline models.

On this line, reversibility is also intriguing and holds promise in addressing some of the limitations and challenges associated with preclinical research. For instance, it provides a framework for evaluating the extent of therapeutic interventions' effectiveness more comprehensively. This resolution is particularly valuable for understanding the trajectory of disease development to optimize treatment. However, it's compulsory to acknowledge that implementing reversibility as a control in animal models may present practical challenges and require careful experimental design. Factors such as the timing and duration of interventions, choice of animal models and selection of appropriate outcome measures must be correctly pondered to ensure the validity and relevance of the findings.

Overall, these advanced technologies have enabled more precise and nuanced investigations, overcoming many limitations associated with traditional animal models and therefore achieving greater fidelity to human biology and pathology. Integrating multiple layers of control in preclinical research holds the potential to enhance the translational significance of experimental findings and accelerate the development of effective therapies for human diseases.

*The orthogonal approach applied to the fictitious X disease works as scholar example of a successful “network” strategy able to overcome the limitations of the commonly used models and investigatory approaches. The implementation of different systems enables to address the same biological question from three different perspectives: space, time and reversibility. It allows to firstly define the temporal susceptibility of excitatory neurons to protein X accumulation, opening to the prospect of a therapeutic intervention aiming at decreasing protein levels early after birth*.

Continued advancements in technology and interdisciplinary collaborations will be essential in unlocking the full potential of these approaches and addressing the intricate complexities of biological mechanisms at the basis of human diseases.

## Author contributions

LB: Conceptualization, Writing – original draft, Writing – review & editing. AS: Conceptualization, Funding acquisition, Supervision, Writing – original draft, Writing – review & editing.
